# Valproate-induced hyperammonemic encephalopathy after aneurysm clipping surgery: a case report with literature review

**DOI:** 10.3389/fmed.2026.1769203

**Published:** 2026-04-01

**Authors:** Maotang Liu, Tianyi Sang, Xiaojie Lu, Bing Li

**Affiliations:** 1 Department of Neurosurgery, Jiangnan University Medical Center, Jiangnan University, Wuxi, China; 2 Department of Neurosurgery, Wuxi No. 2 People’s Hospital, Wuxi, China

**Keywords:** case report, delirium, hyperammonemia, post-aneurysm surgery, valproate

## Abstract

Valproate-induced hyperammonemic encephalopathy (VHE) is a rare but potentially fatal complication of valproate therapy. It is characterized by elevated blood ammonia levels accompanied by central nervous system dysfunction, with clinical manifestations including varying degrees of impaired consciousness, focal neurological deficits, increased seizure frequency, respiratory abnormalities, and metabolic alkalosis. VHE is more common in patients on long-term or high-dose therapy. Although VHE has been frequently reported in neurology and psychiatry, and valproate is widely used for seizure prophylaxis in neurosurgical patients, VHE is easily misdiagnosed or overlooked in the postoperative setting due to the complex etiology of impaired consciousness (e.g., cerebral edema, cerebral infarction, delayed hemorrhage, cerebral vasospasm, seizures, brainstem injury, electrolyte disturbances, and effects of anesthetic agents). We present a case highlighting the need for vigilance regarding VHE and the timely initiation of ammonia-lowering therapy, even when isolated disturbance of consciousness occurs on the fifth postoperative day in the presence of normal liver function.

## 1 Background

Valproate (VPA), as a broad-spectrum antiepileptic drug, is widely used for the prevention and treatment of seizures in neurosurgical patients. While its efficacy is well-established, associated adverse effects cannot be ignored. Among these, Valproate-induced hyperammonemic encephalopathy (VHE) is a rare but potentially life-threatening complication (1, 2). The hallmark of VHE is a significant elevation in blood ammonia levels accompanied by abnormal central nervous system function. Clinical manifestations are diverse, including impaired consciousness, cognitive and behavioral changes, focal neurological deficits, increased seizure frequency, abnormal respiratory patterns, and metabolic alkalosis (3).

Diagnosing VHE is particularly challenging in postoperative neurosurgical patients. The causes of postoperative impaired consciousness are complex and varied, commonly including intracranial factors (such as cerebral edema, intracranial hemorrhage, cerebral infarction, cerebral vasospasm, seizures, intracranial infection) and systemic factors (such as electrolyte imbalances, metabolic abnormalities, and residual effects of anesthetic agents) (4–8). In this context, the clinical symptoms of VHE can be masked by other more common postoperative complications, leading to misdiagnosis or delayed diagnosis, which may exacerbate neurological damage and even be life-threatening.

The pathogenesis of VHE is complex, primarily involving three aspects: First, valproate and its metabolites can inhibit key enzymes in the urea cycle—carbamoyl phosphate synthetase I (CPS1) and N-acetylglutamate synthase (NAGS)—leading to ammonia accumulation in the body (2, 9–11). Second, the drug can cause carnitine depletion, affecting fatty acid β-oxidation, thereby interfering with ammonia metabolism and clearance (9, 12, 13). Additionally, valproate can induce mitochondrial dysfunction, affecting energy metabolism and ammonia detoxification processes, leading to astrocyte swelling, cerebral edema, and neurological symptoms (9, 10, 14, 15). It is noteworthy that VHE can occur in patients with completely normal liver function; therefore, it cannot be ruled out based solely on normal liver function tests.

With increasing clinical awareness, reports of VHE have gradually increased in recent years. Systematic reviews suggest that the overall incidence of VHE in patients taking valproate is approximately 2%–4%, but the risk significantly increases in patients with advanced age, postoperative status, polypharmacy, malnutrition, and other risk factors. Therefore, high vigilance regarding the prevention, recognition, and management of VHE is essential when using valproate in the neurosurgical perioperative period (2, 16, 17).

By reviewing the pathophysiology, clinical manifestations, and diagnostic challenges of VHE, and combining this with a case of VHE occurring after intracranial aneurysm clipping surgery, this article aims to emphasize that blood ammonia monitoring should be routinely performed and VHE included in the differential diagnosis of impaired consciousness for neurosurgical patients receiving valproate, in order to achieve early recognition and timely intervention, thereby improving patient outcomes.

## 2 Literature review of VHE cases after neurosurgery

Although VHE has been reported more frequently in psychiatric and neurological patients on long-term medication, its occurrence in the postneurosurgical context is more insidious and challenging. Table 1 summarizes the key features of published cases of VHE after neurosurgery in recent years. Comprehensive literature shows that postoperative VHE mostly occurs in the early stage (2–7 days) (18) after medication, and it is often seen in high-risk patients such as old age, multi-drug combination, and malnutrition. The main clinical manifestation was disturbance of consciousness, which was easily confused with routine postoperative complications. The core of treatment is to immediately stop VPA and start ammonia lowering therapy. Most patients have a good prognosis. The present case occurred on the fifth postoperative day and the diagnosis was delayed to the eighth day, highlighting the challenge of identifying such complications after aneurysm surgery.

**TABLE 1:** Characteristics of valproate-induced hyperammonemic encephalopathy (VHE) cases reported after neurosurgery.

Author (year)	Number of cases	Surgical type	Time of VHE onset [postoperative day(s)]	Peak blood ammonia (μ mol/L)	Primary treatment	Outcome
Woo et al. (18)	12	Various craniotomies	2–7	120–410	Discontinuation of VPA, supportive care, some with ammonia-lowering drugs	All recovered
Kumarasamy et al. (19)	6	Tumor resection, hematoma evacuation	3–5	89–380	Discontinuation of VPA, L-carnitine	5 recovered, 1 had residual cognitive impairment
Chen et al. (2)	1	Post-traumatic brain injury surgery	4	248.1	Discontinuation of VPA, L-ornithine L-aspartate	Complete recovery
This case (2025)	1	Aneurysm clipping	5	>294	Discontinuation of VPA, switched to LEV, L-ornithine L-aspartate + lactulose	Rapid and complete recovery

## 3 Case introduction

**FIGURE 1:**
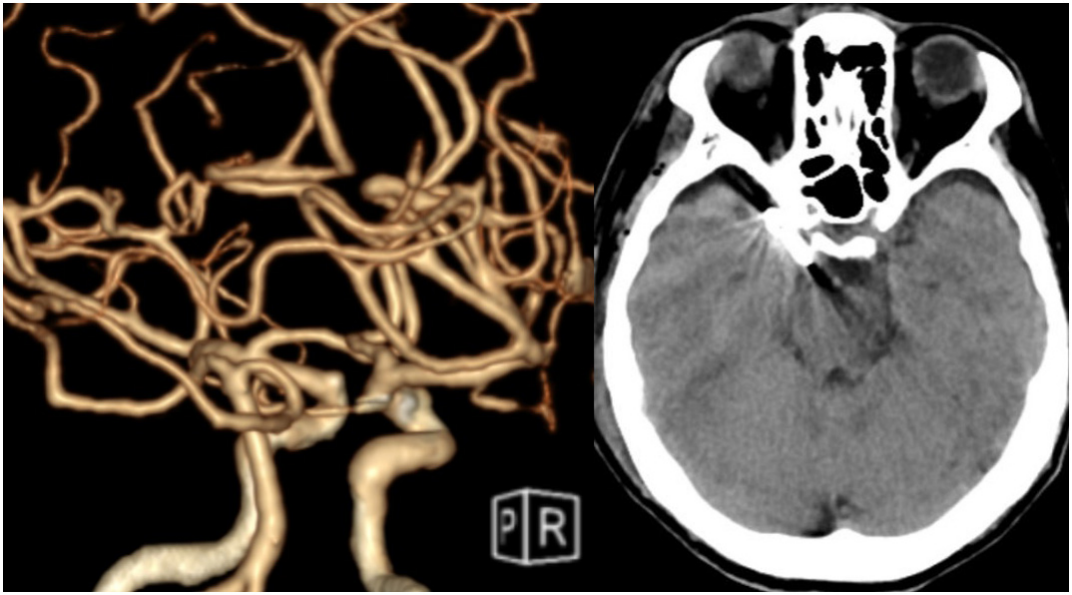
Preoperative cerebral digital subtraction angiography (DSA).

**FIGURE 2:**
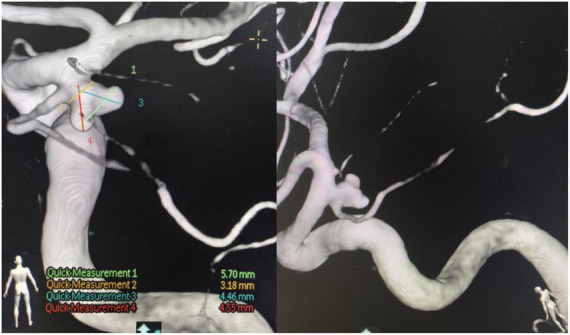
Postoperative non-contrast cranial computed tomography (CT) and CT angiography (CTA).

A 68-year-old male was admitted on 21 June 2025, due to “posterior communicating artery aneurysm found during examination 4 days ago” (Figure 1). He had no history of liver disease, and physical examination on admission revealed no positive signs. On 30 June 2025, he underwent a right pterional keyhole approach craniotomy for clipping of the posterior communicating artery aneurysm under general anesthesia. The procedure was successful (Figure 2). Postoperatively, valproate sodium concentrated injection solution (600 mg, Q12H) was administered intravenously for seizure prophylaxis.

**FIGURE 3:**
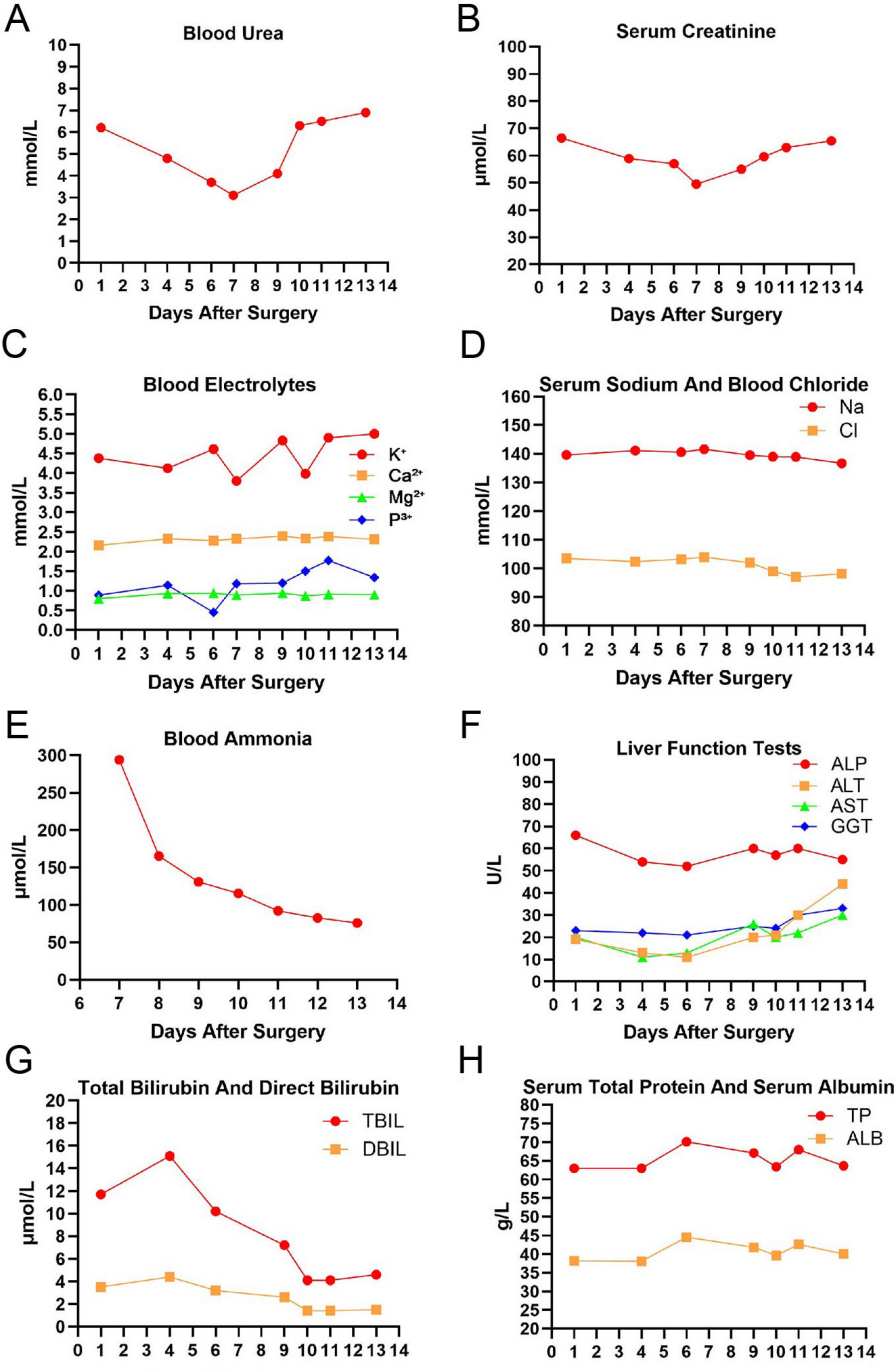
Serial perioperative laboratory trends, including blood ammonia, liver function, renal function, and electrolyte levels. **(A)** Trend of blood urea levels within 14 days. **(B)** Trend of serum creatinine levels within 14 days. **(C)** Trend of blood electrolytes, including potassium (K^+^), calcium (Ca^2 +^), magnesium (Mg^2 +^), and phosphorus (P^*y* +^) levels within 14 days. **(D)** Trend of sodium (Na) and chloride (Cl) levels within 14 days. **(E)** Trend of blood ammonia levels from day 7 to day 14. **(F)** Trend of liver function indicators, including alkaline phosphatase (ALP), alanine aminotransferase (ALT), aspartate aminotransferase (AST), and gamma-glutamyl transferase (GGT). **(G)** Trend of total bilirubin (TBil) and direct bilirubin (DBil) levels within 14 days. **(H)** Trend of total protein (TP) and albumin (ALB) levels within 14 days.

On postoperative day 5, the patient developed progressive impairment of consciousness; by day 6, he became drowsy, and his condition further deteriorated to coma on day 7. During this period, emergency cranial + cervical CTA, cranial MRI plain scan + DWI, and blood biochemistry, liver and kidney function, and blood glucose monitoring were performed, but no definitive cause for the decreased level of consciousness was identified. Blood ammonia was not measured until the early morning of postoperative day 8, when a markedly elevated level (>294.00 μmol/L, exceeding the upper limit of detection) was identified, leading to the diagnosis of hyperammonemic encephalopathy (Figure 3). The following comprehensive treatment measures were immediately initiated. Following this, comprehensive treatment measures were promptly initiated. First, sodium valproate was discontinued and substituted with levetiracetam for antiepileptic therapy. Second, L-ornithine L-aspartate for injection (10 g diluted in 500 mL of 5% glucose solution) was administered via an intravenous infusion pump at a rate of 100 mL/h every 12 h. Third, the patient received oral lactulose solution (20 mL every 8 h) in combination with glycerin enema to manage constipation. Fourth, intravenous human albumin and amino acid infusions were discontinued, while adequate daily intake of calories, fluids, electrolytes, and vitamins was continuously ensured. Finally, serial measurements of serum ammonia levels were performed. After these interventions, physical examination on the morning of postoperative day 8 revealed significant recovery of bilateral corneal reflexes, and repeat serum ammonia levels decreased to 165.6 μmol/L. That afternoon, the patient’s level of consciousness improved from coma to stupor, and he was able to open his eyes in response to verbal stimuli. By postoperative day 9, the patient had regained clear consciousness, with a Glasgow Coma Scale (GCS) score of 14.

During the patient’s care, we systematically ruled out the following common causes: Non-convulsive status epilepticus: Although continuous electroencephalogram monitoring was not performed, the patient did not exhibit limb convulsions, rhythmic eyelid or orofacial movements, and cranial MRI showed no new ischemic or edematous lesions, making non-convulsive status epilepticus less likely. Postoperative cerebral ischemia or hemorrhage: Postoperative MRI did not show new signs of infarction, hemorrhage, or vasospasm. Intracranial infection: The patient had no fever and negative signs of meningeal irritation. Although no lumbar puncture was performed, there was no ventricular dilatation or meningeal enhancement on imaging, and there was insufficient evidence of infection. Metabolic disorders: There were no significant abnormalities in blood electrolytes, blood glucose, liver and kidney function, and arterial blood gases. Finally, a significant increase in blood ammonia (>294 μmol/L) became the key diagnostic basis, and the decrease in blood ammonia after treatment was synchronized with the improvement of consciousness, so VHE was diagnosed.

## 4 Discussion

Valproate (VPA), as a broad-spectrum antiepileptic drug, is widely used for the prevention and treatment of seizures in neurosurgical patients. However, one of its potential adverse effects, hyperammonemic encephalopathy (VHE), although rare, carries a potentially fatal risk and is easily overlooked, especially in postoperative neurosurgical patients (18, 19).

Compared with previous reports, this case enriches the clinical understanding of VHE after neurosurgery in the following aspects: (1) Delayed onset: VHE in this case occurred on the 5th day after surgery, which was later than most cases (2–3 days) after aneurysm surgery, suggesting that drug-related encephalopathy should be considered even within 1 week after surgery. (2) Prominent diagnostic delay: in the complex identification of postoperative disturbance of consciousness, it took 3 days to make a definite diagnosis in this case, highlighting the need for early detection of blood ammonia when routine examinations cannot explain the deterioration of consciousness. (3) Efficacy of combined ammonia-lowering strategy: intravenous infusion of L-Ornithine L-aspartate combined with lactulose can significantly reduce blood ammonia within 24 h and rapidly improve consciousness, which provides a rapid and multi-route treatment reference for acute VHE. (4) This case highlights that normal liver function does not preclude the development of valproate-induced hyperammonemic encephalopathy.

The mechanism by which valproate induces hyperammonemia is complex, primarily involving the following aspects: Urea cycle inhibition: valproate and its metabolites can inhibit key enzymes in the urea cycle—carbamoyl phosphate synthetase I (CPS1) and N-acetylglutamate synthase (NAGS)—leading to ammonia accumulation in the body (20). Carnitine depletion: valproate can cause carnitine deficiency, affecting fatty acid β-oxidation, thereby impeding ammonia metabolism pathways (21). Mitochondrial dysfunction: valproate can interfere with mitochondrial function, affecting energy metabolism and ammonia clearance capacity (22). It is noteworthy that this patient had no history of liver disease, and liver function tests showed no significant abnormalities, indicating that VHE can occur in the context of normal liver function, consistent with literature reports. Therefore, VHE cannot be ruled out based solely on normal liver function.

The etiology of postoperative impaired consciousness in neurosurgical patients is diverse, ranging from intracranial factors (e.g., cerebral edema, hemorrhage, infarction, vasospasm, infection) to systemic factors (e.g., electrolyte disturbances, metabolic acidosis/alkalosis, systemic infection) (4–7). Under these circumstances, VHE may be overlooked. In this case, the patient’s consciousness level decreased on day 6. Initial investigations including head CTA, MRI, and multiple laboratory tests failed to identify a clear cause until elevated blood ammonia was detected on postoperative day 8, confirming the diagnosis. This underscores that blood ammonia should be a routine screening parameter in the differential diagnosis of postoperative impaired consciousness in neurosurgery, especially in patients using valproate.

The clinical manifestations of VHE, as reported previously, mainly include: impaired consciousness (drowsiness, stupor, coma); cognitive and behavioral changes (delirium, disorientation); focal neurological deficits; increased seizure frequency or new types of seizures; abnormal respiratory patterns; and metabolic alkalosis (23, 24). This case presented with typical progressive impairment of consciousness, gradually developing from alertness to drowsiness and coma, consistent with the clinical progression of VHE. Notably, the patient did not exhibit worsened seizures or other focal signs, suggesting that VHE can present as “pure” impairment of consciousness, increasing diagnostic difficulty.

Upon confirming VHE, our team immediately implemented comprehensive treatment measures: prompt discontinuation of valproate, switching to levetiracetam, combined with L-Ornithine L-aspartate intravenous infusion and lactulose for bowel regulation. Blood ammonia decreased from >294 to 165.6 μmol/L within 24 h, accompanied by significant improvement in the patient’s conscious state, demonstrating the favorable effect of early intervention. L-Ornithine L-aspartate is an ammonia-lowering drug (25). Its mechanism is mainly through two pathways to promote ammonia metabolism. On the one hand, ornithine, as a substrate of urea cycle, directly promotes the conversion of ammonia to urea. On the other hand, aspartic acid can promote glutamine synthesis, thereby assisting in the storage and transport of ammonia in the non-toxic glutamine form (26). In this case, given the patient’s extremely high blood ammonia level (>294 μmol per liter) and progression to coma, intravenous infusion was chosen to provide rapid metabolic substrate provision, enhance urea synthesis, and form a multipathway ammonia-lowering strategy with the intestinal deamination of lactulose. Compared with lactulose or L-carnitine alone, ornithine aspartate has a faster onset of action and is especially suitable for the emergency treatment of acute hyperammonemia, which is of great significance in the acute phase management of VHE after neurosurgery.

In recent years, reports of VHE have gradually increased domestically and internationally, but most cases involve long-term medication use in psychiatric or neurological patients, with fewer reports in postoperative neurosurgical settings. This case highlights the importance of preoperative assessment of liver function and metabolic status when valproate is used in the neurosurgical perioperative period. Close postoperative monitoring of consciousness is warranted to detect atypical neurological deterioration. Promptly check blood ammonia levels, especially when impaired consciousness cannot be explained by other causes. Establish a contingency plan for VHE to ensure rapid management upon diagnosis.

Therefore, targeted blood ammonia monitoring is recommended for high-risk populations, including elderly patients (>65 years), long-term bedridden postoperative patients, neurosurgical patients with malnutrition, hepatic or renal insufficiency, and those receiving multiple medications, particularly antiepileptic drugs, antibiotics, and sedatives. In patients without these risk factors, blood ammonia testing should be performed when disturbances of consciousness, behavioral abnormalities, or unexplained neurological deterioration occur, rather than being included as a mandatory routine monitoring parameter.

## 5 Conclusion

Valproate-induced hyperammonemic encephalopathy is a rare but reversible and potentially serious complication in neurosurgical patients. We report a patient who underwent aneurysm clipping and received valproic acid for postoperative seizure prophylaxis, subsequently developing progressive impairment of consciousness on postoperative day 5. Hyperammonemic encephalopathy was confirmed by elevated blood ammonia levels. Following discontinuation of valproic acid and initiation of L-ornithine–L-aspartate in combination with lactulose, the patient’s level of consciousness improved rapidly.

Therefore, routine monitoring of blood ammonia levels should be considered in postoperative patients receiving valproic acid, particularly in high-risk populations such as elderly, long-term bedridden, or malnourished patients, and those receiving multiple medications. In addition, a rapid and standardized diagnostic and therapeutic pathway should be established to facilitate early recognition and timely intervention. In cases of unexplained disturbance of consciousness or neurological deterioration, measurement of blood ammonia may facilitate early identification and timely initiation of ammonia-lowering therapy.

## Data Availability

The original contributions presented in this study are included in this article/supplementary material, further inquiries can be directed to the corresponding authors.

## References

[1] Ma J Fan X Cai X Ji H Li Y Guo J. Effect of ANKK1 polymorphisms on serum valproic acid concentration in chinese han adult patients in the early postoperative period. *Neurol Ther.* (2023) 12:197–209. 10.1007/s40120-022-00419-8 36401149 PMC9837366

[2] Chen W Wang D Fu W Wang J Chen M Li J et al. Valproate-induced hyperammonemic encephalopathy: the role of clinical pharmacists in medication safety-a case report. *J Med Case Rep.* (2025) 19:302. 10.1186/s13256-025-05294-z 40598597 PMC12220738

[3] Kaneko M Shikata H Kihara H. Valproate-Induced hyperammonemic encephalopathy successfully treated with levocarnitine in an elderly patient without liver dysfunction. *Cureus.* (2024) 16:e76603. 10.7759/cureus.76603 39881899 PMC11775636

[4] Akel O Gigliotti MJ Jareczek FJ Zacko JC. Sudden Neurologic Worsening. *Neurosurg Clin N Am.* (2025) 36:433–41. 10.1016/j.nec.2025.03.014 40543951

[5] Wang XJ. Research progress of postoperative delirium in neurosurgery. *World J Psychiatry.* (2025) 15:104708. 10.5498/wjp.v15.i4.104708 40309599 PMC12038677

[6] Sonneville R Benghanem S Jeantin L de Montmollin E Doman M Gaudemer A et al. The spectrum of sepsis-associated encephalopathy: a clinical perspective. *Crit Care.* (2023) 27:386. 10.1186/s13054-023-04655-8 37798769 PMC10552444

[7] Triplett JD Kutlubaev MA Kermode AG Hardy T. Posterior reversible encephalopathy syndrome (PRES): diagnosis and management. *Pract Neurol.* (2022) 22:183–9. 10.1136/practneurol-2021-003194 35046115

[8] Zheng YY Weng XP Fu FW Cao YG Li Y Zheng GQ et al. Cerebrospinal fluid hypovolemia and posterior reversible encephalopathy syndrome. *Front Neurol.* (2020) 11:591. 10.3389/fneur.2020.00591 32655488 PMC7324723

[9] Segura-Bruna N Rodriguez-Campello A Puente V Roquer J. Valproate-induced hyperammonemic encephalopathy. *Acta Neurol Scand.* (2006) 114:1–7. 10.1111/j.1600-0404.2006.00655.x 16774619

[10] Sammar A Tawfik M Fatima F Butler A Aylor-Lee K. Valproate-Induced hyperammonemic encephalopathy causing new-onset seizures. *Cureus.* (2023) 15:e47288. 10.7759/cureus.47288 38021840 PMC10656206

[11] Iqbal K Kummamuru H Dasari N Koritala T Jain NK Deepika K et al. A case of valproic-acid induced hyperammonemic encephalopathy. *Cureus.* (2021) 13:e20380. 10.7759/cureus.20380 35036212 PMC8753585

[12] Farooq F Sahib Din J Khan AM Naqvi S Shagufta S Mohit A. Valproate-Induced hyperammonemic encephalopathy. *Cureus.* (2017) 9:e1593. 10.7759/cureus.1593 29062625 PMC5650254

[13] Verrotti A Trotta D Morgese G Chiarelli F. Valproate-induced hyperammonemic encephalopathy. *Metab Brain Dis.* (2002) 17:367–73. 10.1023/a:1021918104127 12602513

[14] Levy A Very E Montastruc F Birmes P Jullien A Richaud L. Case report: a case of valproic acid-induced hyperammonemic encephalopathy associated with the initiation of lithium: a re-duplicable finding. *Front Psychiatry.* (2022) 13:875636. 10.3389/fpsyt.2022.875636 35586415 PMC9108155

[15] Wu MY Chang FY Ke JY Chen CS Lin PC Wang TS. Valproic acid-induced hyperammonemic encephalopathy in a patient with bipolar disorder: a case report. *Brain Sci.* (2020) 10:187. 10.3390/brainsci10030187 32213827 PMC7139302

[16] Wesselman K Cavaliere V Goyal R Anderson E. Valproate, risperidone, and paliperidone: a case of valproate-induced hyperammonemic encephalopathy. *Ment Health Clin.* (2024) 14:28–32. 10.9740/mhc.2024.02.028 38312439 PMC10836567

[17] Huang TK Su YC Shih CS. Valproic acid-induced hyperammonemia with encephalopathy in adults: a meta-analysis. *Int J Clin Pharmacol Ther.* (2025) 63:105–13. 10.5414/CP204673 39873559

[18] Woo PYM Woo AWY Lam SW Ko NMW Ho JWK Chu ACH et al. Incidence, presentation, and risk factors for sodium valproate-associated hyperammonemia in neurosurgical patients: a prospective, observational study. *World Neurosurg.* (2020) 144:e597–604. 10.1016/j.wneu.2020.09.027 32916358

[19] Kumarasamy S Basheer N Raheja A Tandon V Kumar Laythalling R Kale SS. Valproate-induced hyperammonemic encephalopathy in neurosurgical patients: our experience and systematic literature review. *Neurosurg Rev.* (2024) 47:836. 10.1007/s10143-024-03054-z 39503745

[20] Aires CC van Cruchten A Ijlst L de Almeida IT Duran M Wanders RJ et al. New insights on the mechanisms of valproate-induced hyperammonemia: inhibition of hepatic N-acetylglutamate synthase activity by valproyl-CoA. *J Hepatol.* (2011) 55:426–34. 10.1016/j.jhep.2010.11.031 21147182

[21] McCarron EP. Valproate induced carnitine deficiency and hyperammonaemia. *Clin Med.* (2023) 23:429. 10.7861/clinmed.Let.23.4.1 37524421 PMC10541038

[22] Moedas MF Simões RJM Silva MFB. Mitochondrial targets in hyperammonemia: addressing urea cycle function to improve drug therapies. *Biochem Pharmacol.* (2024) 222:116034. 10.1016/j.bcp.2024.116034 38307136

[23] Sousa C. Valproic acid-induced hyperammonemic encephalopathy - a potentially fatal adverse drug reaction. *Springerplus.* (2013) 2:13. 10.1186/2193-1801-2-13 23451336 PMC3579419

[24] Chopra A Kolla BP Mansukhani MP Netzel P Frye MA. Valproate-induced hyperammonemic encephalopathy: an update on risk factors, clinical correlates and management. *Gen Hosp Psychiatry.* (2012) 34:290–8. 10.1016/j.genhosppsych.2011.12.009 22305367

[25] Jain A Sharma BC Mahajan B Srivastava S Kumar A Sachdeva S et al. L-ornithine L-aspartate in acute treatment of severe hepatic encephalopathy: a double-blind randomized controlled trial. *Hepatology.* (2022) 75:1194–203. 10.1002/hep.32255 34822189

[26] Goh ET Stokes CS Sidhu SS Vilstrup H Gluud LL Morgan MY. L-ornithine L-aspartate for prevention and treatment of hepatic encephalopathy in people with cirrhosis. *Cochrane Database Syst Rev.* (2018) 5:CD012410. 10.1002/14651858.CD012410.pub2 29762873 PMC6494563

